# A Reference Hand-Book of the Medical Sciences

**Published:** 1887-11

**Authors:** 


**A Reference Hand-Book of the Medical Sciences, embracing the Entire Range of Scientific and Practical Medicine and Allied Science.** Illustrated by chromo-lithograph and fine wood engravings. Edited by Albert H. Buck, M. D. Vol. V. New York: Wm. Wood & Co., 56 Lafayette place. 1887.

The fifth volume of this magnificent work has come to hand. It covers the subjects which, under the alphabetical arrangement followed, fall between Mil. and Pot. In every respect, this volume equals its predecessors. The illustrations are many of them new, and are sufficiently abundant. The subjects are treated, in almost every case, in a way which shows the hand of a master, and a reference to the author usually gives the name of some distinguished writer. The complete work bids fair to be the most valuable compendium of the science of medicine, especially for the practitioner, ever issued in any language.

